# Recurrent urticaria with fever, arthralgia, and biclonal gammopathy

**DOI:** 10.1016/j.jdcr.2024.08.037

**Published:** 2024-09-19

**Authors:** Vijay K. Somani, Ashwini Annabathula, Anirudh Somani, Indukooru S. Reddy

**Affiliations:** a Department of Dermatology, Dermatrendz, Hyderabad, Telangana, India; b Department of Dermatology, Dermacare, Hyderabad, Telangana, India; c Department of Dermatology, Apollo Hospital, Hyderabad, Telangana, India

**Keywords:** colchicine, IgA gammopathy, IgG gammopathy, kappa and lambda chains, Schnitzler syndrome

## Case history

**Fig 1:**
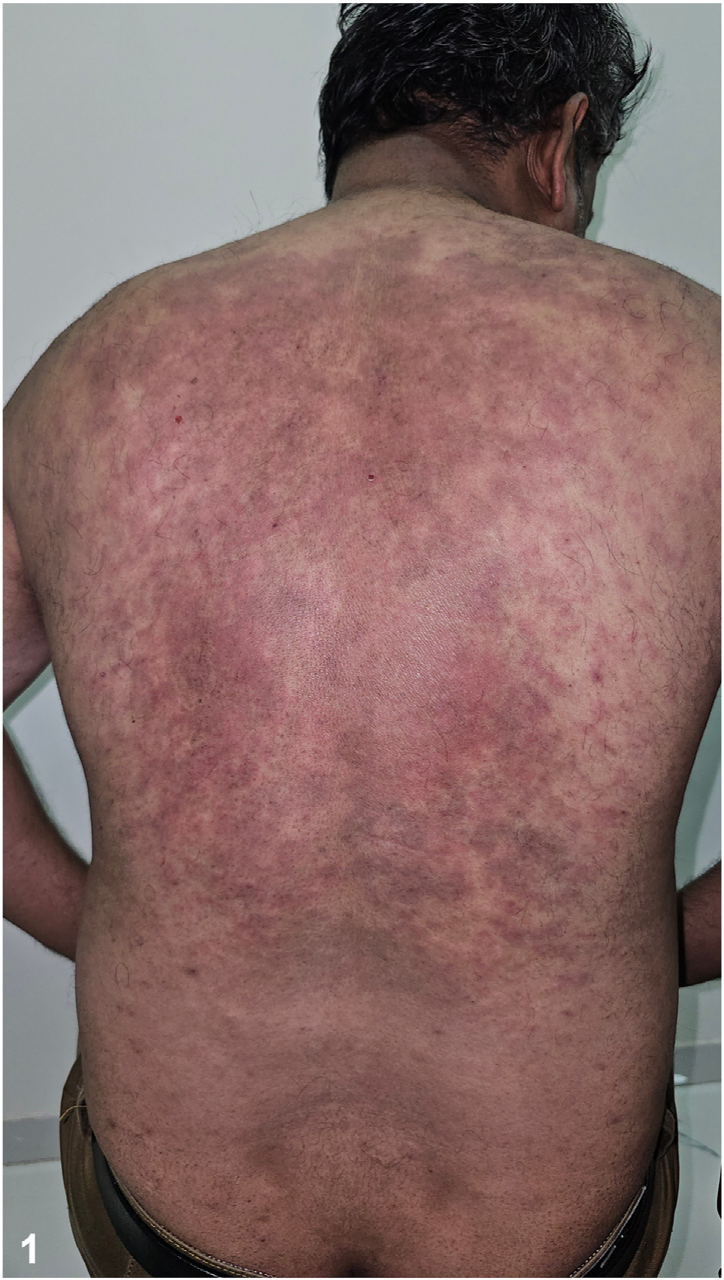
▪▪▪

**Fig 2:**
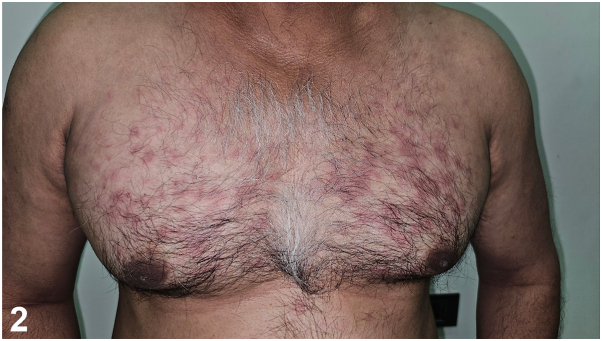
▪▪▪

A 47-year-old man gave a history of recurrent episodes of fever, joint pains, bone pains, and a rash over trunk and arms since 3 years. Examination revealed, multiple erythematous circular/polycyclic patches, 2 to 5 cm, lasting for about 24 hours (Figs 1 and 2). Investigations revealed, increased C reactive protein and erythrocyte sedimentation rate levels. Immunoglobulin (Ig) assay showed high IgA (353.5 mg/dL), and increased IgG (2135 mg/dL). Serum complement and ferritin levels were normal. Serum protein electrophoresis revealed biclonal gammopathy involving IgG and IgA with increased κ and λ chains. Patient declined bone marrow examination. Histopathology showed superficial and deep perivascular mixed infiltrate predominantly of neutrophils without vasculitic changes.

**Question 1: What is the most likely diagnosis?**

A. Monoclonal gammopathy of undetermined significance
B. Adult-onset Still disease
C. Schnitzler syndrome (SS)
D. Urticarial vasculitis
E. Cryopyrin associated periodic syndromes

**Answers:**

A. Monoclonal gammopathy of undetermined significance – Incorrect. This very common plasma cell dyscrasia is characterized by the presence of a monoclonal Ig in the serum or urine. It is typically asymptomatic and is seen in older individuals. This patient presented with fever, urticaria, and constitutional symptoms.
B. Adult-onset Still disease – Incorrect. It presents with a spiking fever, evanescent rash with high serum ferritin levels and elevated hepatic transaminases. This patient, on the other hand, had episodes of recurrent urticaria, lasting about 24 hours, and a nonspiking fever associated with biclonal gammopathy involving both IgG and IgA.
C. Schnitzler syndrome (SS) – Correct. The Strasbourg criteria^1^ for diagnosis requires 2 major criteria - recurrent, nonpruritic urticaria, and monoclonal gammopathy, and 3 minor criteria, which include recurrent fever, objective findings of abnormal bone remodeling with or without bone pain, neutrophilic dermal infiltrate on skin biopsy, and elevated CRP and/or leukocytosis. This patient fulfilled Strasbourg criteria: 2 major criteria, recurrent urticaria and gammopathy, and 3 minor criteria, fever, raised ESR and CRP levels, and neutrophilic urticarial histopathology.
D. Urticarial vasculitis – Incorrect. Presentation is similar to SS but the histopathology should reveal features of true vasculitis with fibrinoid necrosis of small vessel walls, and also may show complement consumption. Both these features were absent in the present case.
E. Cryopyrin associated periodic syndromes – Incorrect. Cryopyrin associated periodic syndromes including neonatal onset multisystem inflammatory disease, Muckle-Wells syndrome, and familial cold autoinflammatory syndrome generally present at an earlier age and are not associated with a serum monoclonal protein.

**Question 2: Which of the following is true with regard to SS?**

A. Diagnosis can be confirmed by specific tests
B. Most frequently seen in third and fourth decade
C. IgG monoclonal gammopathy with increased λ light chains is characteristically seen in SS in >80% of cases
D. SS can progress to lymphoreticular malignancy in 15% to 20% of cases
E. Histopathology typically shows vasculitic changes with fibrinoid necrosis and leukocytoclasis with nuclear dust

**Answers:**

A. Diagnosis can be confirmed by specific tests – Incorrect. There is no specific diagnostic test for SS and is usually diagnosed by excluding other conditions presenting with a similar picture. Lipsker or Strasbourg criteria have been proposed and are very helpful in clinical practice to confirm the diagnosis. The 2 obligate criteria being recurrent non-pruritic urticaria and monoclonal gammopathy and minor criteria (2 criteria in IgM gammopathy and 3 in IgG gammopathy) such as fever, bone pains, increased inflammatory markers, leukocytosis, and the histology.
B. Most frequently seen in third and fourth decade – Incorrect. Typically, patients are in the sixth decade of their life at diagnosis and mean age of disease onset is 51 years.
C. IgG monoclonal gammopathy with increased λ light chains is characteristically seen in SS in >80% of cases – Incorrect. IgM monoclonal is present in the majority of the patients (94%) with κ light chain overwhelmingly involved (85%), and IgG gammopathy comprises of a minority (6%).^2^
D. SS can progress to lymphoreticular malignancy in 15% to 20% of cases – Correct. Most of the patients have a chronic benign course. Progression to lymphoreticular malignancy determines the prognosis in SS. Lymphoma or Waldenstrom disease occurs in 15% to 20% of patients.^3^^,^^4^ Other less frequently associated lymphoproliferative disorders include lymphoplasmacytic lymphoma, chronic lymphocytic leukemia, multiple myeloma, and marginal zone B-cell lymphoma.^5^
E. Histopathology typically shows vasculitic changes with fibrinoid necrosis and leukocytoclasis with nuclear dust – Incorrect. The histologic picture seen in SS is typically described as a neutrophilic urticarial dermatosis, and constitutes one of the minor criterion in Strasbourg diagnostic criteria. There is perivascular and interstitial neutrophilic infiltrate without changes associated with vasculitis. No evidence of vasculitis distinguishes SS from urticarial vasculitis. Lack of dermal edema helps in excluding Sweet syndrome.

**Question 3: Which agents have been successfully used in the treatment of SS?**

A. Tumor necrosis factor α inhibitors
B. Interleukin (IL) 1 inhibitors
C. IL-6 inhibitors
D. B and C
E. Rituximab

**Answers:**

A. Tumor necrosis factor α inhibitors – Incorrect. Tumor necrosis factor inhibitors including etanercept, adalimumab, and infliximab have been tried and generally are ineffective.
B. Interleukin (IL) 1 inhibitors – Correct. This group of medications are most successful in the management of SS. Anakinra is the most commonly prescribed, followed by canakinumab and rilonacept. Anakinra, a recombinant IL-1RA, has quick onset of action and is most effective in the majority of patients treated and shows sustained long-term efficacy. Both canakinumab, human monoclonal antibody against IL-1β and rilonacept, a recombinant fusion protein, acting as a decoy receptor, are effective in SS but are less beneficial than anakinra.
C. IL-6 inhibitors – Correct. Tocilizumab, an IL-6 inhibitor is useful in SS, but tends to lose efficacy over time. It has been tried successfully in patients not responding to anti–IL-1 therapy.
D. B and C – Correct. Both the IL-1 and IL-6 inhibitors have been used successfully in SS. No other treatment modality offers comparable results. Corticosteroids, immunosuppressants, thalidomide, colchicine, dapsone, etc have been used with little or no efficacy.
E. Rituximab – Incorrect. Rituximab, a chimeric monoclonal antibody against CD20 has been tried in some cases, with mixed results.

## Conflicts of interest

None disclosed.
